# Psychometric validation of the Chinese version of the Short Inflammatory Bowel Disease Questionnaire and evaluation of its measurement invariance across sex

**DOI:** 10.1186/s12955-021-01890-x

**Published:** 2021-11-07

**Authors:** Dajuan Sun, Lili Chi, Jiahui Liu, Junwei Liang, Song Guo, Shaojie Li

**Affiliations:** 1 First Clinical Medical College, Shandong University of Traditional Chinese Medicine, Jinan, 250355 China; 2 Department of Spleen and Stomach Diseases, Affiliated Hospital of Shandong University of Traditional Chinese Medicine, Jinan, 250014 China; 3 Department of Social Medicine and Health Service Management, Xiangya School of Public Health, Central South University, Changsha, 410078 China

**Keywords:** Inflammatory bowel disease, Quality of life, Assessment tool, Validation, Reliability, Measurement invariance

## Abstract

**Background:**

This study aimed to evaluate the psychometric properties of the Chinese version of the Short Inflammatory Bowel Disease Questionnaire (C-SIBDQ), and its measurement invariance across sex in Chinese patients with inflammatory bowel disease (IBD).

**Methods:**

Between September 2018 and July 2021, 284 patients with IBD were recruited from a spleen and stomach clinic. All participants completed the C-SIBDQ, 12-item Short-Form Health Survey (SF-12), nine-item Patient Health Questionnaire Depression Scale (PHQ-9), and the seven-item Generalized Anxiety Disorder Scale (GAD-7). Floor and ceiling effects were evaluated by testing frequencies and composition ratios for the minimum and maximum C-SIBDQ scores. Exploratory and confirmatory factor analysis (CFA) were used to evaluate the C-SIBDQ’s factor structure and construct validity. Convergent validity was evaluated through examining bivariate correlations between the C-SIBDQ and the SF-12, PHQ-9, and GAD-7. Internal consistency reliability and retest reliability were evaluated by respectively calculating the Cronbach’s α and the intraclass correlation coefficient (ICC) among a subsample (n = 79) after 2 weeks. The measurement invariance across sex was evaluated through multiple-group CFA.

**Results:**

The C-SIBDQ scores showed no floor or ceiling effects and had a single-factor structure and good convergent validity, with significant correlations with the SF-12, PHQ-9 and GAD-7. Good internal consistency (Cronbach’s α = 0.920) and test–retest reliability (ICC = 959) were observed. The C-SIBDQ also showed measurement invariance across sex, and females showed higher C-SIBDQ scores than males.

**Conclusions:**

The C-SIBDQ has high reliability, validity, and stability across sex, and can be used in clinics to assess the health-related quality of life of patients with IBD.

## Background

Inflammatory bowel disease (IBD) represents a group of chronic nonspecific intestinal inflammatory diseases with unclear etiology, including ulcerative colitis (UC) and Crohn’s disease (CD) [1]. In China, there were approximately 350,000 IBD cases in total between 2005 and 2014; however, according to the Chinese Center for Disease Control and Prevention, this figure is expected to reach 1.5 million by 2025 [2]. IBD is characterized as a long-term disease with difficult treatment and easy susceptibility to relapse; it also imposes serious economic pressure, medical burden, and psychological burden for patients, their family caregivers, and society [3], and has become a major public health problem that needs to be solved urgently.

Health-related quality of life (HRQoL) refers to one’s perception of their physical and mental well-being and the effect of disease and/or its treatment on this perception [4]. Recently, an obvious trend has been observed regarding the more frequent usage of patient HRQoL assessments as outcome indicators for chronic conditions, including IBD [5]. A previous systematic review demonstrated that the Inflammatory Bowel Disease Questionnaire (IBDQ) developed by Guyatt et al. [6], which is widely used to assess disease-specific HRQoL among patients with IBD, is one of the most reliable and valid evaluation tools in this regard. The IBDQ comprises 32 items and four dimensions: intestinal symptoms, systemic symptoms, social function, and emotional function [7]. The IBDQ has been psychometrically validated among Chinese populations [8]. However, it should be noted that, IBDQ administration is relatively time-consuming (approximately 20 min) due to its large number of items, [9]; thus, respondents are prone to fatigue and poor compliance. From the perspective of survey efficiency, questionnaire-design experts generally believe that short, simple, and easy-to-answer questionnaires are most appropriate, as they can reduce the burden on the respondents and surveyors [10].

In an attempt to improve the efficiency of the survey and reduce its burden, Irvine et al. [11] developed a short version of the IBDQ (known as the “SIBDQ”); consequently, they found that the SIBDQ has high reliability and validity. Patients who are fully competent in terms of reading and writing English can complete the SIBDQ in approximately 5 min. The SIBDQ comprises 10 items and contains the same four dimensions as the IBDQ [11]; thus, it can rapidly evaluate the physical, social, and emotional status in patients with IBD. It has been widely used worldwide, both in clinical practice and academic research. However, further cross-cultural verification and psychometric validation is required in non-English speaking countries, since SIBDQ was originally developed in English. German [12] and Portuguese [13] versions of SIBDQ have been successfully verified and have reported good reliability and validity. However, to the best of our knowledge, there has been no psychometric validation of the Chinese version of the SIBDQ (C-SIBDQ). A previous China-based study used the SIBDQ to investigate the HRQoL of patients with IBD during the Coronavirus Disease 2019 pandemic [14]. It is not feasible to use the C-SIBDQ without conducting a psychometric validation, as it is not clear whether its assessment of HRQoL among Chinese populations is valid and credible. Verification of the C-SIBDQ would help to ensure the standardization of assessment results and make them more scientific and reliable.

Measurement invariance is an important index that reflects the validity of a scale; it refers to measuring whether a tool has the same structure and/or meaning across different groups (e.g., between males and females) or across different time points [15]. Previous studies have shown statistically significant differences between males and females regarding SIBDQ scores [16]. However, it is not clear whether this difference is due to sex or the structure of the measurement instrument; moreover, existing studies have not examined the measurement invariance of the SIBDQ across sex. In order to accurately compare the measurement results of a self-report tool across multiple groups, it is essential to establish the cross-group measurement invariance of the scale [17]. Exploring the measurement invariance of the SIBDQ would be beneficial for improving the accuracy of HRQoL assessments of patients with IBD and ensuring comparability between subgroups.

Thus, the objectives of the present study are to evaluate, in the context of Chinese patients with IBD, the psychometric properties of the C-SIBDQ and its measurement invariance across sex.

## Methods

### Participants

From September 2018 to July 2021, a total of 284 patients with IBD were recruited from a spleen and stomach clinic in Jinan, China. Convenience sampling was used to select participants from among the patients attending the clinic. The inclusion criteria were: (1) clinically diagnosed with IBD; (2) aged 18 years or older; (3) willing to voluntarily participate in the survey (after being fully appraised of the study content and goals); and (4) able to read and understand all of the questionnaire content. The exclusion criterion was having a severe cognitive impairment such as dementia. Two weeks after the initial sample had completed the study questionnaire, 78 members of the sample completed the C-SIBDQ retest.

This study was ethically approved by the Medical Ethics Committee of the Affiliated Hospital of Shandong University of Traditional Chinese Medicine (Identification Code: 2017-010-KY). All participants provided written informed consent.

### Measures

#### Demographic and clinical characteristics questionnaire

The participants were administered a questionnaire that collected demographic and clinical characteristics, including age, sex, residence, education level, marital status, type of IBD, and disease activity.

The Mayo score [18] was used to assess the disease activity of UC. The Mayo score included four clinical indicators: bowel frequency, rectal bleeding, endoscopy results, and overall doctor evaluation. Each indicator was rated using a 4-point Likert scale (0–3 points), and the total score ranged from 0 to 12. The scores were categorized as remission (0–2), mild (3–5), and moderate (6–10) and severe (11–12).

The Crohn's Disease Activity Index (CDAI) [19] was used to assess the disease activity of CD. CDAI comprised eight scoring indicators: the number of loose stools, the number of days of abdominal pain, general health, extraintestinal manifestations and complications, opioid antidiarrheals, abdominal masses, hematocrit reduction, and standard weight deviation. The total score was obtained by computing the weighted sum of all the eight indicators scores, ranging from 0 to 600 points. The CDAI scores were categorized as remission (< 150), mild (150–219), and moderate (220–450) and severe (> 450), respectively.

#### SIBDQ

The original SIBDQ is a self-report questionnaire that comprises 10 items and measures four dimensions: bowel symptoms, emotional function, systemic symptoms, and social function. For each item, respondents are asked to indicate, in regard to the past 2 weeks, their IBD symptoms and the impact of IBD on their overall feelings and mood. Each item is scored using a 7-point Likert scale (ranging from 1 to 7), with total scores ranging from 10 to 70; the higher the score, the better the respondent’s HRQoL. In this study, considering that the Chinese version of the IBDQ has been verified and is widely used in China [20, 21], for the present investigation we directly used all 10 items from the existing C-SIBDQ, without further translation.

#### 12-item Short-Form Health Survey

The 12-item Short-Form Health Survey (SF-12) is a universal assessment tool for quality of life [22], and has previously been found to have good reliability and validity among patients with IBD [23]. The scale contains 12 items and two components: physical health and mental health. The total score is determined by converting the sum of the item scores to a 0–100-point range using a standardized method [24]. The higher the score, the better the respondent’s quality of life. In this study, the Cronbach's α coefficient for this scale was 0.874. We used the SF-12 as an evaluation indicator of the convergent validity of the C-SIBDQ.

#### 9-item Patient Health Questionnaire Depression Scale

The 9-item Patient Health Questionnaire Depression Scale (PHQ-9) is a tool for measuring depressive symptoms and was compiled based on the nine diagnostic criteria of depression stipulated in the Diagnostic and Statistical Manual of Mental Disorders, fourth edition (DSM-IV) [25]. It has been validated among patients with IBD [26]. The scale contains nine items, and each item is scored using a 4-point Likert-type scale (0 = “*nothing at all*,” 3 = “*every day*”). The higher the score, the more severe the respondent’s depressive symptoms. For this study, the Cronbach’s α coefficient for the scale was 0.917. The PHQ-9 was used as an evaluation indicator of the convergent validity of the C-SIBDQ.

#### 7-item Generalized Anxiety Disorder Scale

The 7-item Generalized Anxiety Disorder Scale (GAD-7) is a tool for measuring generalized anxiety disorder based on the diagnostic criteria for anxiety disorder stipulated in the DSM-IV [27]. It has been validated among patients with IBD [28]. The scale contains seven items, and each item is scored using a 4-point Likert-type scale (0 = “*nothing at all*,” 3 = “*every day*”). The higher the score, the more severe the respondent’s generalized anxiety disorder. For this study, the Cronbach’s α coefficient for this scale was 0.951. The GAD-7 was used as an evaluation indicator of the convergent validity of the C-SIBDQ.

### Data analysis

SPSS version 25 (IBM SPSS Statistics, Armonk, NY, USA) was used to perform descriptive analysis, Student’s *t*-test, Pearson’s correlation analysis, exploratory factor analysis (EFA), and an internal consistency test. Mplus 7.0 was used to perform confirmatory factor analysis (CFA). Continuous variables, such as C-SIBDQ scores, were described using means ± standard deviations (SDs), and categorical variables, such as sex, were described using n (%).

Floor and ceiling effects were evaluated based on the frequencies and composition ratios of the minimum and maximum C-SIBDQ scores. Minimum and maximum composition ratios of less than 15%, respectively, were considered to indicate an absence of floor or ceiling effects [29]. Reliability was evaluated using internal consistency and intraclass correlation coefficient (ICC) in the overall and retest sample, respectively. Validity evaluation was computed using construct and convergence validity. Construct validity was evaluated through factor analysis. We randomly divided the total sample into two groups: sample A (n = 142; 86 males, 56 females) and sample B (n = 142; 89 males, 53 females). Through a chi-squared test and Student’s *t*-test, we determined that sample A and B did not statistically significantly differ in terms of demographic characteristics, clinical characteristics, or C-SIBDQ scores. Sample A was used for EFA. The Kaiser–Meyer–Olkin (KMO) measure of sampling adequacy and Bartlett’s test of sphericity were used to assess the EFA suitability of the C-SIBDQ data [30, 31]; when the KMO value is greater than 0.50 and Bartlett’s sphericity test is significant, the data are considered suitable for factor analysis [31]. Sample B was used for CFA with maximum likelihood estimation. We used chi-square value/degrees of freedom (χ^2^/df), comparative fit index (CFI), the Tucker-Lewis index (TLI), and root mean square error of approximation (RMSEA) to evaluate the CFA model fit. A χ^2^/df of less than 5.000, CFI and TLI of greater than 0.900, and an RMSEA of less than 0.080 indicate good model fit [32].

Multiple-group CFA was used to assess the C-SIBDQ’s measurement invariance across sex. First, we constructed factor structure models of the C-SIBDQ for male (n = 175) and female (n = 109) samples, respectively. Second, we constructed a configural invariance model to evaluate whether the factor structures for the male and female samples were consistent. Third, we constructed a metric invariance model by limiting factor loading. Finally, on the basis of this limited factor loading, we constructed a scalar invariance model by limiting the intercept. If the changes in the fitting indices CFI (ΔCFI) and RMSEA (ΔRMSEA) from the configural invariance model to the metric invariance model to the scalar invariance model were less than 0.010 [33], this would indicate that the C-SIBDQ has measurement invariance. In this study, the significance level of all hypothesis tests was less than 0.05.

## Results

### Participants’ demographic and clinical characteristics

The final sample included 284 patients with IBD (249 with UC, 35 with CD). Their mean age was 41.63 ± 11.88 years (range: 19–79 years). Most were male (61.6%), lived in urban areas (67.3%), had university or higher education level, and were married (88.0%). The average duration of disease was 5.54 ± 5.57 years. The mean scores for the SF-12, PHQ-9, and GAD-7 were 59.55 ± 20.27, 9.10 ± 6.25, and 7.05 ± 5.51, respectively. More details are shown in Table 1.

**Table 1:** Descriptive statistics for the participants

Variables	Total sample (N = 284)
	N (%)/M ± SD
*Age (years)*	41.63 ± 11.88
18–44	172 (60.6)
45–59	85 (29.9)
≥ 60	27 (9.5)
*Sex*	
Males	175 (61.6)
Females	109 (38.4)
*Residence*	
Urban area	191 (67.3)
Rural area	93 (32.7)
*Educational level*	
Primary school and below	16 (5.6)
Junior high school	53 (18.7)
High school	60 (21.1)
University and above	155 (54.6)
*Marital status*	
Married	250 (88.0)
Unmarried	34 (12.0)
*Type of IBD*	
Ulcerative colitis	249 (87.7)
Crohn’s disease	35 (12.3)
*Disease activity*	
Remission	87 (30.6)
Mild	74 (26.1)
Moderate	79 (27.8)
Severe	44 (15.5)
C-SIBDQ	46.96 ± 12.52
SF-12	59.55 ± 20.27
PHQ-9	9.10 ± 6.25
GAD-7	7.05 ± 5.51

IBD, Inflammatory Bowel Disease, C-SIBDQ, Chinese version of the Short Inflammatory Bowel Disease Questionnaire, SF-12 = Short-Form Health Survey, PHQ-9, Patient Health Questionnaire Depression Scale, GAD-7, Generalized anxiety disorder scale

### Score distribution of the C-SIBDQ

The mean, SD, skewness, kurtosis, and minimum and maximum values for the C-SIBDQ were 46.96, 12.52, − 0.60, − 0.24, 14.00, and 70.00, respectively. All skewness and kurtosis values were between − 1 and 1, indicating that the C-SIBDQ data in this study followed normal univariate distribution. The number of participants, who scored minimum and maximum values, were two and three, respectively, giving constituent ratios of 0.7% and 1.1%, respectively; this indicated that this scale had no floor or ceiling effects.

### Construct validity

EFA and CFA were used to evaluate the construct validity of the C-SIBDQ. First, based on Sample A, we conducted the KMO test and Bartlett’s test of sphericity. The KMO value was 0.917, and the Bartlett’s test of sphericity statistic was 799.136 (*P* < 0.001); this indicated that the C-SIBDQ was suitable for factor analysis. Second, we conducted principal component analysis with orthogonal rotation on the 10 items; consequently, one factor was extracted with eigenvalues of 5.641, indicating that it explained 56.4% of the total variance. All items had factor loadings of 0.557 or larger on this factor (Table 2). Third, using the data for Sample B, we performed CFA on the single-factor structure obtained through EFA. The CFA results showed that the single-factor model had a good fit to the data (χ^2^/df = 28.759/22 = 1.307, RMSEA = 0.047, CFI = 0.993, TLI = 0.985). Therefore, according to the EFA and CFA results, the C-SIBDQ showed a single-factor structure.

**Table 2:** Loadings of each item on single-factor model for C-SIBDQ in EFA and CFA

Items	Sample A (N = 142)	Sample B (N = 142)
	Factor loading	Factor loading
Item 1	0.557	0.684
Item 2	0.758	0.806
Item 3	0.837	0.785
Item 4	0.867	0.905
Item 5	0.850	0.739
Item 6	0.680	0.722
Item 7	0.683	0.656
Item 8	0.600	0.591
Item 9	0.815	0.758
Item 10	0.793	0.796

### Convergent validity

The results of the Pearson’s correlation analysis illustrated that the C-SIBDQ is positively correlated with SF-12 scores (*r* = 0.790, *P* < 0.001), and negatively correlated with PHQ-9 (*r* =  − 0.675, *P* < 0.001) and GAD-7 scores (*r* =  − 0.628, *P* < 0.001).

### Internal consistency and test–retest reliability

The internal consistency of the C-SIBDQ was good (Cronbach’s α was 0.920). High test–retest reliability (ICC = 0.959) was found for a sub-sample (n = 78) measured 2 weeks later.

### Measurement invariance across sex

The measurement invariance of the C-SIBDQ across sex was also tested. The results of the CFA for the male and female samples showed that the model fit the data well, indicating that multiple-group CFA was appropriate. In addition, the fit indices of the configural invariance model, metric invariance model, and scalar invariance model were satisfactory. When comparing the metric invariance model with the configural invariance model and the scalar invariance model, both ΔRMSEA and ΔCFI were less than 0.010. This indicated that the C-SIBDQ has measurement invariance across sex. Table 3 shows the fit indices for all measurement invariance models and the inter-model differences.

**Table 3:** Fit indices of all measurement invariance model and inter-model differences of the C-SIBDQ

Model	χ^2^	df	χ^2^/df	RMSEA	CFI	TLI	△RMSEA	△CFI
Males	25.058	22	1.139	0.028	0.997	0.993		
Females	34.347	22	1.561	0.072	0.985	0.970		
Configural invariance model	59.404	44	1.350	0.050	0.991	0.982		
Metric invariance model	75.655	53	1.427	0.055	0.987	0.978	0.005	0.004
Scalar invariance model	85.568	62	1.380	0.052	0.987	0.981	0.003	< 0.001

### Sex difference in the C-SIBDQ

The *t*-test results showed a statistically significant sex difference in C-SIBDQ scores (*t* = 2.591, *P* = 0.010), with females (mean = 49.39, SD = 12.07) showing higher scores than males (mean = 45.46, SD = 12.59).

## Discussion

This study verified the reliability and validity of the C-SIBDQ and its measurement invariance across sex. To the best of our knowledge, this study is the first to evaluate the psychometric properties and measurement invariance of the C-SIBDQ among Chinese patients with IBD. The results showed that the C-SIBDQ is suitable for this population and has good psychometric characteristics. This means that the C-SIBDQ is a relatively short and easy-to-use tool that community and clinical staff can utilize to assess the HRQoL of patients with IBD in China.

The C-SIBDQ showed no floor or ceiling effects, indicating sufficient reactivity and content validity [34]. In addition, our study showed that a newly established single-factor structure is the most suitable factor structure for the C-SIBDQ; the C-SIBDQ is inconsistent with the four-factor structure of the original SIBDQ [11] and the versions in other languages [13]. The previous four-factor structure comprised intestinal symptoms, social function, emotional function, and systemic symptoms, which provides a detailed reflection of HRQoL for certain populations [11]. However, from the perspective of psychosomatic medicine, the physical and psychological conditions of a disease are interrelated and influenced by social culture [35]. This means that social culture may induce factor differences. For example, although both the original version of the SIBDQ and the Portuguese version contain a four-factor structure [11, 13], there are differences between the two versions regarding which items are assigned to each factor; this is because differing languages and cultural backgrounds can affect patients’ understanding of the SIBDQ [13]. The difference in the factor structure does not affect the application of the C-SIBDQ because all 10 items showed good factor loadings and were retained. Moreover, the score for the C-SIBDQ was determined to be related to the scores for the SF-12, PHQ-9, and GAD-7, showing good convergent validity; this finding is similar to those of previous validation studies [13]. Further, a previous study found that the active inflammatory state of IBD intersects with the pathobiology of depression and anxiety [36], while a questionnaire-based study also found depression and anxiety to relate to the disease activity of IBD and the HRQoL of patients with IBD [37]. Both of these studies support our above-mentioned results concerning convergent validity.

Our research showed that the C-SIBDQ’s Cronbach’s α and ICC were 0.920 and 0.959, respectively, indicating good internal consistency reliability and test–retest reliability. This result is similar to those for the Portuguese and German versions [12, 13]. In the verification of the Portuguese version, Cronbach’s α and ICC were both determined to be 0.80, while in the German version, the SIBDQ’s Cronbach’s α and ICC were determined to be 0.84 and 0.60, respectively. However, compared to these findings, the C-SIBDQ reports higher reliability. In addition, the reliability measurement result for the C-SIBDQ is consistent with the levels observed in previous China-based surveys that used other evaluation tools (e.g., the IBDQ) [20].

We verified the newly established single-factor structure’s measurement invariance (configuration, metric, scalar) for male and female samples. The results showed that the C-SIBDQ can reliably account for differences between male and female patients. This is certainly important, because it has previously been reported that there are differences in SIBDQ scores between sex groups [16]. Our study also showed that females (mean = 49.39, SD = 12.07) have higher C-SIBDQ scores than males (mean = 45.46, SD = 12.59). Therefore, measurement invariance across sex was supported; that is, the difference in C-SIBDQ scores reflected the true difference between males and females.

This verification of the C-SIBDQ has important practical significance for patients with IBD, caregivers, and medical staff. First, its low number of items and easy-to-use format can afford quick evaluations of the HRQoL of patients with IBD in clinical environments, which can help medical staff understand patients’ problems and implement targeted treatment. Second, in relation to clinical intervention research, researchers can use it to evaluate the effectiveness of treatment or nursing measures. Third, patients can use the C-SIBDQ for self-assessment in order to determine their own HRQoL and whether they require medical attention. Caregivers can also provide diet management and psychological support to patients based on the results of the C-SIBDQ.

### Limitations

This study has the following limitations. First, we only recruited participants from one clinic; thus, the results may not be representative of all patients with IBD in China. In the future, a more representative sample should be used to verify the reliability and validity of the C-SIBDQ. Second, the number of UC and CD patients in our study was not balanced; we did not group these for verification because there were fewer CD patients than UC patients. In the future, in order to verify the applicability of the C-SIBDQ for UC and CD populations, respectively, and compare the two, it will be necessary to collect larger sample sizes from multiple clinics. Third, this study did not investigate other disease-related factors affecting HRQoL among patients with IBD, such as the course of disease, comorbidities, and extraintestinal diseases. These disease factors should be considered when evaluating HRQoL in IBD patients in the future.

### Implications for future studies

With the development of clinimetrics, more and more scholars believe psychometrics is restricted as it: evaluates component homogeneity, directs insufficient attention towards sensitivity, and insufficiently evaluates clinical utility [38–40]. Clinimetrics aims to evaluate the sensitivity, clinical utility and validity of the patient-related scale from a clinical perspective [41, 42]. The systematic review suggests that psychometrics and clinical measurement should be combined in the development and verification of patient -related scales to make sure that the scales are credible and effective in both psychological and clinical measurements [43]. Therefore, future studies need to explore the clinical characteristics of C-SIBDQ further. Future researchers can analyze the sensitivity of C-SIBDQ and its ability to distinguish between the HRQoL of patients with different disease characteristics (such as disease course), according to the clinimetric criteria for patient-reported outcome measures [38]. In addition, the predictive effectiveness of C-SIBDQ—whether it can predict the future development or recurrence of IBD—can also be examined.

## Conclusions

The C-SIBDQ has high reliability, validity, and stability across sex, and can be used in clinics as a tool for assessing the quality of life of patients with IBD.

## Data Availability

The datasets used and/or analyzed during the current study are available from the corresponding author via email.

## Abbreviations

IBD: Inflammatory bowel disease
UC: Ulcerative colitis
CD: Crohn’s disease
HRQoL: Health-related quality of life
IBDQ: Inflammatory Bowel Disease Questionnaire
SIBDQ: Short Inflammatory Bowel Disease Questionnaire
C-SIBDQ: Chinese version of the Short Inflammatory Bowel Disease Questionnaire
SF-12: 12-Item Short-Form Health Survey
PHQ-9: 9-Item Patient Health Questionnaire Depression Scale
GAD-7: 7-Item Generalized Anxiety Disorder Scale
ICC: Intraclass correlation coefficient
EFA: Exploratory factor analysis
CFA: Confirmatory factor analysis
SD: Standard deviation
KMO: Kaiser–Meyer–Olkin
CFI: Comparative fit index
TLI: Tucker–Lewis index
RMSEA: Root mean square error of approximation
